# Evaluation of the efficacy of Er,Cr:YSGG laser in Treating oral benign soft tissue lesions

**DOI:** 10.34172/joddd.40905

**Published:** 2024-12-14

**Authors:** Zainab Fadhil Odah, Hanan Jafer Taher, Ammar Saleh AlAlawi

**Affiliations:** ^1^Medical and Biological Applications Branch, Institute of Laser for Postgraduate Studies, University of Baghdad, Baghdad, Iraq; ^2^Photonics Units, Institute of Laser for Postgraduate Studies, University of Baghdad, Baghdad, Iraq; ^3^Laser Surgery Unit/Department of Maxillofacial Surgery/Al-Emamein Al-Kadhemein Medical City, Baghdad, Iraq

**Keywords:** Er,Cr:YSGG, Fibroepithelial polyp, Laser, Oral soft tissue lesions, Pyogenic granuloma

## Abstract

**Background.:**

Managing benign oral soft tissue lesions presents a clinical challenge, often requiring invasive procedures with associated drawbacks. The Er,Cr:YSGG laser has emerged as a potential solution, given its precision and minimal tissue disruption in other medical contexts.

**Methods.:**

This pre-post study involved 16 participants diagnosed with benign oral soft tissue lesions. The lesions were excised using the Er,Cr:YSGG laser (2780 nm) with 2.75‒3.75-W power outputs. Pain was quantified using a numerical rating scale from 0 to 10, and edema presence was noted. Function interference and satisfaction were graded. Healing was assessed via the Early Wound Healing Score (EHS). The observation spanned three postoperative intervals: day 1, day 3, and day 7.

**Results.:**

This study included 16 participants with benign oral soft tissue lesions, predominantly pyogenic granuloma and fibroepithelial polyp. Initial discomfort and bleeding were common, while a small proportion reported pain. Er,Cr:YSGG laser treatment significantly reduced discomfort (VAS score) and promoted healing (EHS score) over time. Function interference decreased, and patient satisfaction improved from day 1 to day 7. Additionally, bleeding scores decreased drastically, confirming the laser’s effective hemostatic properties.

**Conclusion.:**

The findings suggest that Er,Cr:YSGG laser treatment presents a promising, less invasive approach for managing oral benign soft tissue lesions, prioritizing patient well-being and contentment alongside successful lesion removal.

## Introduction

 The oral cavity is a common site for a diverse range of tumors and tumor-like lesions, which can be benign or malignant. Factors such as inadequate oral hygiene, tobacco use, denture placement, and certain habits can contribute to the development of reactive lesions and tumors. Making an accurate diagnosis requires a complete clinical history and awareness of the symptoms and signs, including the location of the oral mucosal lesion and its size, color, and morphology. However, the definitive diagnosis is confirmed through histopathologic analysis of the affected tissue. Clinicians may reduce dentoalveolar effects by promptly identifying and treating such lesions.^1,2^

 Oral health is vital for the quality of life of all individuals. Oral lesions can cause discomfort or pain that interferes with mastication, swallowing, and speech. They also produce symptoms such as halitosis, xerostomia, or oral dysesthesia, which interfere with daily social activities.^3^

 Benign neoplasms and hyperplastic tumorous lesions are prevalent in oral pathology, marked by slow growth and distinct borders, detectable upon palpation while lacking discoloration or ulceration. Typically painless, these lesions often remain stable over extended periods. They commonly occur on the buccal mucosa, particularly along the occlusion line, tongue, alveolar process, and palate—especially in removable denture-bearing regions. The predominant structural element comprises hyperplastic fibrous connective tissue coated with hyperplastic or acanthotic epithelium. This composition increases vulnerability to bacterial and fungal infections. These lesions can be removed by different surgical procedures such as the conventional scalpel, electrosurgical scalpel, or lasers.^4,5^

 The increasing adoption of lasers in medicine is driven by their advantageous properties, including efficient coagulation that reduces postoperative bleeding and diminishes pain and edema. Furthermore, lasers facilitate effective and prompt healing, resulting in minimal discomfort during and after interventions and rapid resolution of symptoms.^6,7^

 Er,Cr:YSGG laser, belonging to the erbium family, is classified as a hard tissue laser with a specific wavelength of 2780 nm. This laser system comprises yttrium, scandium, gallium, and garnet (YSGG), doped with erbium and chromium. One of its noteworthy attributes is its strong absorption by water, a characteristic that facilitates its application on soft tissues without inducing undesirable thermal damage. This property has led to its effective use in medical procedures involving oral benign soft tissue lesions.^8,9^

 While conventional methods for managing benign oral soft tissue lesions exist, there is a growing interest in exploring advanced techniques that offer enhanced precision, reduced patient discomfort, and improved esthetic outcomes. The Er,Cr:YSGG laser has shown promising potential in various medical applications, including soft tissue ablation. However, its specific efficacy and safety profile in treating benign oral soft tissue lesions require rigorous investigation. This study explored the effectiveness of the Er,Cr:YSGG laser in the excision of various oral soft tissue lesions by measuring pain, swelling, bleeding, and healing.

## Methods

###  Study design

 This study, which ran from January 1st, 2023, to September 1, 2023, used a pre-post interventional design. Using a convenience sampling technique, 16 participants (11 females and 5 males) with exophytic soft tissue lesions were included in the study. They were 11‒62 years old. Histopathological analysis using an excisional biopsy technique was used to diagnose the benign lesion. Some patients were treated at the Laser Medical Research Clinic of the University of Baghdad, while others were treated at Nawar Mousa Specialty Dental Center.

###  Laser system

 The Er,Cr:YSGG laser system (Waterlase iPlus, Biolase, California, USA) employed in this work had the following characteristics: 2780-nm wavelength, power ranging from 2.75 to 3.75 W, a pulse rate of 50 Hz, air at 20%, and water at 40%. The MGG6 tip in S mode was employed, with the laser tip maintained in direct contact with the oral lesion.

###  Surgical procedure

 Before the surgical intervention, a comprehensive medical and dental history was collected, encompassing any relevant information.

 Before the operation began, local anesthesia was achieved using 2% lidocaine with 1:180 000 adrenaline. The length of the surgical procedure varied from 10 to 15 minutes for each patient. After administering the local anesthetic agent, the lesion or tumor was excised from its base using tissue forceps. After carefully positioning the laser handpiece perpendicular to the surgical site, the excision procedure was started from the pedicellar base of the mass and continued in all directions until the tumor was completely removed. Finally, coagulation (laser bandage) was achieved by setting the laser at 0.5 W, 1% water, and 20% air, and the tip in non-contact mode. The wounds were healed by secondary intention after the surgical treatment.

 After being quickly fixed in a 10% formalin solution, all the excised biopsies were sent for histopathological analysis to determine the type of lesions. Analgesics were given to patients as needed after surgery to ensure their comfort and pain control during the healing period.

 A numerical rating scale was used to quantify pain, with scores ranging from 0 to 10. The presence of edema was observed and noted. A grading system was used to assess function interference: 0 meant no interference, 1 meant mild, 2 meant moderate, and 3 meant severe interference. Likewise, there were four categories for satisfaction: poor, fair, good, and excellent. The satisfaction categories were identified by assigning a numerical satisfaction score between 0 and 3. Bleeding was further classified into four categories:

- 0: no bleeding
- 1: mild bleeding
- 2: moderate bleeding
- 3: severe bleeding

Healing progression was evaluated using the Early Wound Healing Score (EHS) (Table 1), a validated metric that gauges healing dynamics.^10^ The observation timeline comprised three postoperative intervals: day 1, day 3, and day 7.

**Table 1:** Early wound healing score (EHS)

**Parameter**	**Description **	**Points**
CSR	Merged incision margins	6
	Incision margins in contact	3
	Visible distance between incision margins	0
CSH	Absence of fibrin on the incision margins	2
	Presence of fibrin on the incision margins	1
	Bleeding at the incision margins	0
CSI	Absence of redness along the incision length	2
	Redness involving < 50% of the incision length	1
	Redness involving > 50% of the incision length and/or pronounced swelling	0

Abbreviations: CSR, clinical signs of re-epithelization; CSH, clinical signs of hemostasis; CSI, clinical signs of inflammation.

###  Statistical analysis

 Depending on whether the distribution was normal or skewed, continuous variables were expressed as means and standard deviations or medians with ranges. Categorical variables were expressed as frequencies and percentages. One-way repeated-measures ANOVA (for parametric variables) and Friedman’s rank sum test (for non-parametric variables) were used to test the differences in means and medians over the follow-up periods. Similarly, Cochran’s Q test was used for dichotomous variables. The satisfaction and function levels were recorded as (0,1,2,3) to allow the non-parametric Friedmans’ ANOVA to test the difference between medians. *A* P value < 0.05 was considered statistically significant. R software packages (dplyr, gt_summery and ggplot) were used for data processing, visualization, and statistical analysis (R version 4.3.1, R Foundation for Statistical Computing, Vienna, Austria).

## Results

**Figure 1:**
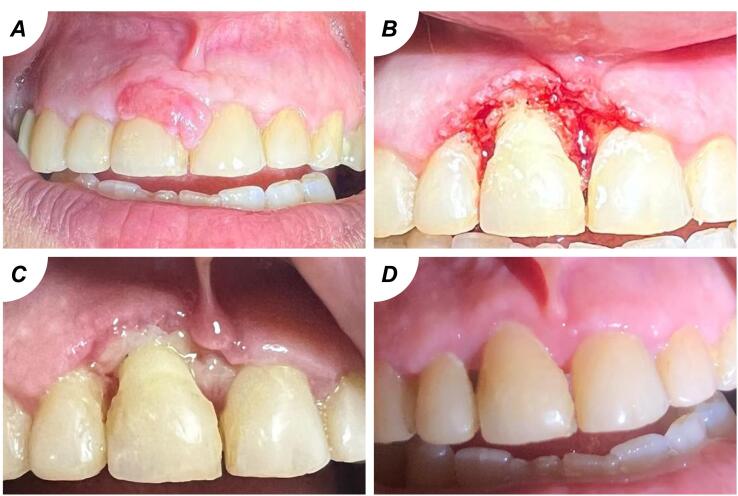
(A) pyogenic granuloma; (B) after the excision; (C) after 7 days; (D) after 1 month

**Figure 2:**
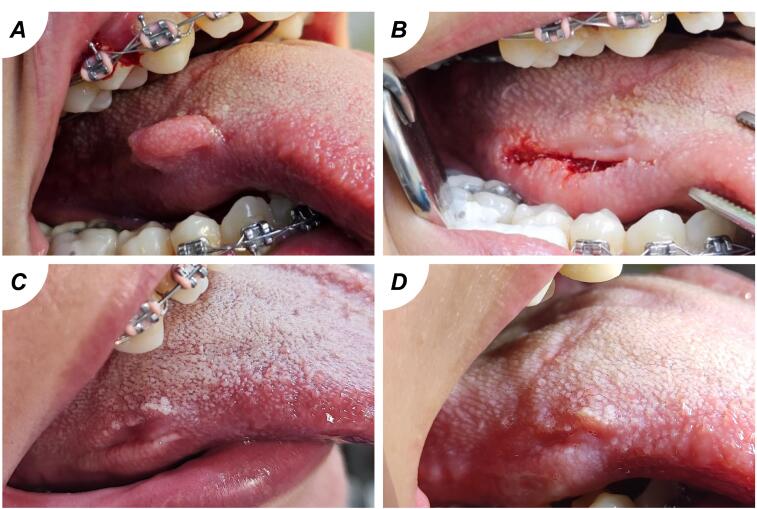
(A) fibroepithelial polyp; (B) after the excision; (C) after 7 days; (D) after 2 weeks

**Figure 3:**
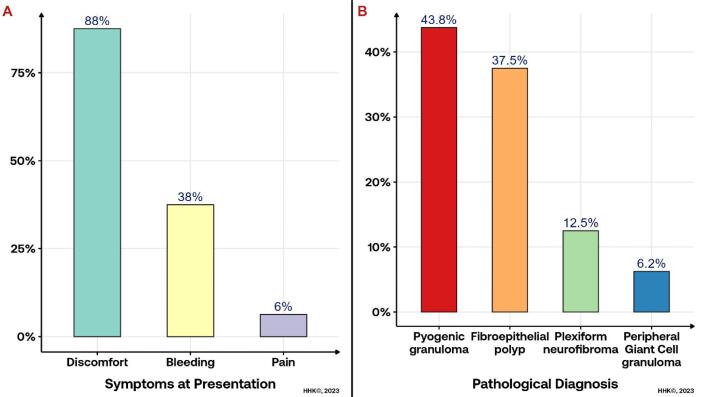
(A) proportion of symptoms; (B) pathological diagnosis of the soft tissue lesion

 The study investigated 16 individuals with benign oral soft tissue lesions. The mean participant age was 41.6 years, predominantly female (68.8%). Occupations varied, with 31.3% being employees and housewives each, followed by 18.8% students and unemployed individuals (Table 2). Predominant diagnoses were pyogenic granuloma (43.8%) (Figure 1) and fibroepithelial polyp (37.5%) (Figure 2). Plexiform neurofibroma and peripheral giant cell granuloma were less common at 12.5% and 6.3%, respectively (Figure 3B). Most experienced discomfort (87.5%), while bleeding was reported by 37.5%, and a minority felt pain (6.3%) (Figure 3A).

**Table 2:** Description of Patient’s demographics

**Characteristic**	**N=16**
Age (y), Mean ± SD	41.6 ± 17.0
Sex, No. (%)	
Female	11 (68.8)
Male	5 (31.3)
Occupation, No. (%)	
Employee	5 (31.3)
Housewife	5 (31.3)
Student	3 (18.8)
Unemployed	3 (18.8)

**Figure 4:**
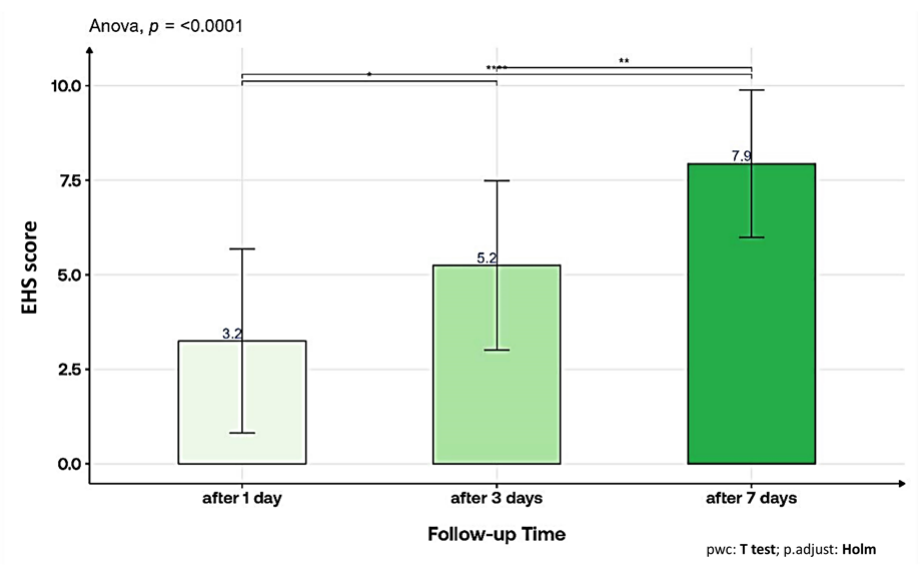
Post hoc analysis of the early wound healing score (EHS) in the study cohort

 The results revealed significant trends in the characteristics observed across different time intervals (Table 3). On day 1, the visual analog scale (VAS) score was 3.0 ± 1.7, indicating initial discomfort, which significantly decreased to 0.8 ± 1.0 on day 3 and reached 0.0 ± 0.0 by day 7 (*P* < 0.001). Edema was present in 6.3% of cases on days 1 and 3, decreasing to 0.0% by day 7 (*P* = 0.367). Notably, the EHS score showed a consistent increase from day 1 (3.2 ± 2.5) to day 7 (7.9 ± 1.9) (*P* < 0.001), reflecting improved healing and tissue recovery (Figure 4). Similar patterns were observed in clinical signs of re-epithelization (CSR), clinical signs of hemostasis (CSH), and clinical signs of Inflammation (CSI) scores, all showing statistically significant improvements over the observation period (*P* < 0.001 or *P* = 0.0007). In terms of function interference, there was a significant shift toward less interference over time (*P* = 0.0023). Patient satisfaction levels showed an increasing trend from day 1 to day 7, with 81.3% reporting “excellent” satisfaction by day 7 (*P* = 0.223). These findings underscore the intervention’s positive impact on pain reduction, tissue healing, and overall patient satisfaction during the study period.

**Table 3:** Description of pain, edema, healing score and quality of life over-the follow-up periods

**Characteristics**	**Day 1**	**Day 3**	**Day 7**	***P*** **value**^a^
VAS score, Mean ± SD	3.0 ± 1.7	0.8 ± 1.0	0.0 ± 0.0	< 0.001
Presence of oedema	1 (6.3%)	1 (6.3%)	0 (0.0%)	0.367
EHS score, Mean ± SD	3.2 ± 2.5	5.3 ± 2.2	7.9 ± 1.9	< 0.001
CSR	1.5 ± 1.2	3.0 ± 1.9	4.9 ± 1.5	< 0.001
CSH	0.8 ± 0.6	1.2 ± 0.4	1.6 ± 0.5	< 0.001
CSI	0.9 ± 0.6	1.1 ± 0.3	1.5 ± 0.5	0.0007
Function interference, No. (%)				0.0023
No	0 (0.0%)	2 (12.5%)	4 (25.0%)	
Mild	9 (56.3%)	11 (68.8%)	10 (62.5%)	
Moderate	6 (37.5%)	2 (12.5%)	1 (6.3%)	
Severe	1 (6.3%)	1 (6.3%)	1 (6.3%)	
Satisfaction, No. (%)				0.223
Good	3 (18.8%)	2 (12.5%)	1 (6.3%)	
Excellent	13 (81.3%)	14 (87.5%)	15 (93.8%)	

Abbreviations: EHS: early wound healing score; CSR: clinical signs of re-epithelization; CSH: clinical signs of hemostasis; CSI: clinical signs of inflammation. ^a^ Repeated Measure ANOVA (Type III test); Cochran Q test; Friedman's ANOVA

 The study showed a significant reduction in bleeding scores following Er,Cr:YSGG laser treatment for oral benign soft tissue lesions (Table 4). Initial mean bleeding scores of 0.8 ± 0.9 decreased substantially to 0.0 ± 0.0 after 3 and 7 days, highlighting the procedure’s effective hemostatic properties (*P* < 0.001, Friedman’s ANOVA).

**Table 4:** Description of bleeding over-the follow-up periods

**Bleeding score**	**Mean+SD**	**Median (range)**	***P*** **value**^a^
Before treatment	0.8 ± 0.9	0.0 (0.0-2.0)	< 0.001
Immediately post-operatively	0.8 ± 0.6	1.0 (0.0-2.0)	
After 1 day	0.3 ± 0.4	0.0 (0.0-1.0)	
After 3 days	0.0 ± 0.0	0.0 (0.0-0.0)	
After 7 days	0.0 ± 0.0	0.0 (0.0-0.0)	

^a^ Friedman's ANOVA.

## Discussion

 Er,Cr:YSGG lasers are becoming increasingly favored in different dental disciplines because of their adaptability. They can be easily applied in both hard and soft tissue procedures, with the ability to adjust settings to match the specific requirements of each treatment.^11^ The Er,Cr:YSGG laser stands out as a highly precise ablation tool with distinct benefits. It exhibits strong absorption by water and results in minimal harm to nearby tissues, particularly the underlying muscular layers. This minimal tissue trauma contributes to a favorable postoperative healing process characterized by minimal scar formation.^12^ Also, this laser minimizes thermal artifacts for pathological examination, seals lymphatics and nerve endings, and provides excellent surgical margins. Other benefits include less bleeding and scarring and a better recovery period with reduced discomfort and swelling.^13^

 The current study indicates significant changes over time in many parameters using the Er,Cr:YSGG laser to manage pyogenic granuloma, fibroepithelial polyp, plexiform neurofibroma, and giant cell granuloma. After a significant reduction in pain, there was no pain on the seventh day. In one instance of pyogenic granuloma, edema occurred but vanished by the seventh day. EHS improvements over time suggest enhanced healing. CSR, CSH, and CSI all showed similar improvements, pointing to promising trends in tissue recovery. Function interference considerably decreased with time, and patient satisfaction levels increased by day 7, with the majority of cases reporting “Excellent” satisfaction. These results demonstrate how well the intervention decreased pain, promoted healing, and increased patient satisfaction throughout the research.

 Comparing our study results with previous research, notably Kumar and colleagues’^11^ case series, there is a consistent theme of the Er,Cr:YSGG laser’s advantages in soft tissue procedures (mucocele excision, pyogenic granuloma excision, maxillary frenectomy, gingival fibroma excision, exposure of unerupted teeth, and lingual frenectomy). Kumar et al^11^ found that the laser reduced the need for local analgesia, shortened procedure times, provided hemostasis, enhanced surgical visibility, and minimized scarring, all beneficial in pediatric cases. Sarkar et al^14^ reported similar advantages with no patient discomfort, no anesthesia requirement, minimal bleeding, and good wound healing.

 Chawla et al^12^ observed favorable outcomes in the case of pyogenic granuloma, with minimal scarring and uneventful healing. Additionally, Eroglu et al^15^ compared Er,Cr:YSGG laser to scalpel in epulis fissuratum removal, emphasizing superior clinical outcomes and reduced analgesic and local anesthetic usage, corroborating the benefits of laser surgery.

 In keeping with this body of evidence, our study emphasizes the effectiveness of the Er,Cr:YSGG laser in reducing pain, edema, and bleeding, promoting healing and generally improving patient outcomes in treating benign oral soft tissue lesions. These consistent results across various soft tissue operations prove the laser’s utility in modern clinical practice. Er,Cr:YSGG lasers reduce pain perception because they can block sensory nerve terminals.^16^ The absence of edema when using the Er,Cr:YSGG laser is explained by the laser-tissue interaction. The increased temperature of the laser causes water molecules to evaporate, creating the thermomechanical effect. This decreases inflammation and edema because there is less damage to the surrounding tissue.^17^

 The findings of our study regarding minimal bleeding immediately after Er,Cr:YSGG laser procedures align with the observations made by Selvaganesh et al,^18^ who reported limited hemostasis and coagulation effect when using Er,Cr:YSGG laser because its primary chromophore in soft tissues is water. Nevertheless, it is important to remember that the Er,Cr:YSGG laser has specialized applications and is unsuitable for treating vascular lesions since hemoglobin cannot be taken in it.

 Conversely, Sarkar et al^19^ investigated using the Er,Cr:YSGG laser to treat oral leukoplakia and reported a different outcome. They observed profuse bleeding during the procedure, which contradicts our study’s findings of minimal bleeding. This discrepancy could be attributed to the nature of the treated lesions, as oral leukoplakia may exhibit different vascular characteristics compared to our study’s benign soft tissue lesions. Furthermore, the wavelength and settings of the Er,Cr:YSGG laser in the two studies might have been adjusted differently, affecting its hemostatic performance. In summary, our study favors minimum bleeding with Er,Cr:YSGG laser for particular lesions; however, it is essential to consider the specific clinical conditions and laser settings when interpreting these findings.

## Conclusion

 In conclusion, the study results indicate that Er,Cr:YSGG laser therapy is a promising and minimally invasive option for treating benign oral soft tissue lesions. It offers enhanced patient comfort and satisfaction while ensuring effective lesion removal. However, further research with larger sample sizes and longer follow-up periods is warranted to validate these findings and establish the long-term efficacy and safety of this approach.

## Competing Interests

 No conflict of interest exists.

## Ethical Approval

 Ethical and scientific approval for the research was obtained from the scientific committee at the University of Baghdad, Institute of Laser for Postgraduate Studies (No:1549, project No. 43 on 25/1/2023). The present study adhered to ethical standards set by the institutional or national research committee, the 1964 Declaration of Helsinki, and its subsequent amendments for all procedures involving human participants. Verbal and written consent was obtained from all patients before starting data collection and after explaining the aims of the study and assuring confidentiality.The ethical approval code is1549, project No. 43 on 25/1/2023.
